# β-catenin Is Essential for Efficient *In Vitro* Premyogenic Mesoderm Formation but Can Be Partially Compensated by Retinoic Acid Signalling

**DOI:** 10.1371/journal.pone.0057501

**Published:** 2013-02-27

**Authors:** Jacob Wong, Virja Mehta, Anastassia Voronova, Josée Coutu, Tammy Ryan, Michael Shelton, Ilona S. Skerjanc

**Affiliations:** Department of Biochemistry, Microbiology and Immunology, Faculty of Medicine, University of Ottawa, Ottawa, Ontario, Canada; University of Minnesota Medical School, United States of America

## Abstract

Previous studies have shown that P19 cells expressing a dominant negative β-catenin mutant (β-cat/EnR) cannot undergo myogenic differentiation in the presence or absence of muscle-inducing levels of retinoic acid (RA). While RA could upregulate premyogenic mesoderm expression, including Pax3/7 and Meox1, only Pax3/7 and Gli2 could be upregulated by RA in the presence of β-cat/EnR. However, the use of a dominant negative construct that cannot be compensated by other factors is limiting due to the possibility of negative chromatin remodelling overriding compensatory mechanisms. In this study, we set out to determine if β-catenin function is essential for myogenesis with and without RA, by creating P19 cells with reduced β-catenin transcriptional activity using an shRNA approach, termed P19[shβ-cat] cells. The loss of β-catenin resulted in a reduction of skeletal myogenesis in the absence of RA as early as premyogenic mesoderm, with the loss of Pax3/7, Eya2, Six1, Meox1, Gli2, Foxc1/2, and Sox7 transcript levels. Chromatin immunoprecipitation identified an association of β-catenin with the promoter region of the *Sox7* gene. Differentiation of P19[shβ-cat] cells in the presence of RA resulted in the upregulation or lack of repression of all of the precursor genes, on day 5 and/or 9, with the exception of Foxc2. However, expression of Sox7, Gli2, the myogenic regulatory factors and terminal differentiation markers remained inhibited on day 9 and overall skeletal myogenesis was reduced. Thus, β-catenin is essential for *in vitro* formation of premyogenic mesoderm, leading to skeletal myogenesis. RA can at least partially compensate for the loss of β-catenin in the expression of many myogenic precursor genes, but not for myoblast gene expression or overall myogenesis.

## Introduction

During embryogenesis, skeletal muscle derives from epithelial structures called somites [1]. Signals emanate from the neural tube, the notochord and the surface ectoderm during somitogenesis inducing the formation and patterning of somites, leading to the dermomyotome and myotome. Wnt signals are amongst the signals secreted from the axial structures during skeletal myogenesis and are sufficient to induce myogenesis in somitic tissue *in vitro* [2]. Mice lacking Wnt1 or Wnt3a fail to form the medial compartment of the DM, and have reduced Pax3 and Myf5 expression [3], [4]. Thus Wnt signalling is important for muscle development in the embryo.

The most well-studied mechanism of Wnt signaling occurs via the canonical Wnt signalling pathway (reviewed in [5]). Wnt ligands bind to Frizzled (Fz) cell surface receptors initiating a signalling cascade that regulates the nuclear translocation of the transcription factor and cell adhesion molecule, β-catenin. In the absence of Wnt signals, β-catenin is marked for degradation through the ubiquitination pathway by interacting with the destruction complex. In the presence of Wnt, the formation of the destruction complex is inhibited and intracellular levels of β-catenin increase, allowing entry into the nucleus and enhanced transcription mediated by interaction with T-cell factors (TCFs) or lymphocyte enhancer factors (LEFs) [5].

The P19 embryonal carcinoma (EC) cell line is a mouse embryonic stem cell (mES) model system [6]. Studies involving the injection of these cells into mouse blastocysts have shown that P19 cells can develop into normal tissues in adult mice, although the mice have defects, including tumours [7]. P19 cells can be induced to form mesoderm through cell aggregation, with the upregulation of Brachyury T by day 3 [8]. Aggregation in the presence of 0.5–1% dimethyl sulfoxide (DMSO) can induce premyogenic mesoderm formation by days 4–5, myoblast formation by days 8–9 and terminal differentiation into skeletal muscle by day 9 [9]. The differentiation of P19 cells into skeletal muscle utilizes similar molecular pathways as those regulating mES and human ES cells [10], [11], [12]. Thus, P19 cells are a good model for *in vitro* skeletal myogenesis, providing a drug-inducible differentiation system which is ideal for examining molecular pathways.

Studies in P19 cells have shown that either Wnt3a or β-catenin can induce skeletal myogenesis, implicating an important role for canonical Wnt [13]. Furthermore, skeletal myogenesis is inhibited in cells overexpressing a dominant negative mutant β-catenin, created by replacing transcriptional activation domain of β-catenin with an Engrailed-2 transcriptional repressor domain, termed β-cat/EnR [13]. Wnt3a acting through β-catenin initiated skeletal myogenesis by stimulating the expression of skeletal muscle progenitor genes, such as Meox1, Gli2, Pax3/7, Six1, Foxc1 and Foxc2 [12], [13], [14] which are genes expressed in the developing somites/premyogenic mesoderm [15], [16], [17], [18], [19], [20]. Knockdown of these genes in the developing embryo results in defects in somitic differentiation/formation or muscle development [16], [21], [22], [23], [24], [25]. Thus, premyogenic mesoderm is specified to the skeletal muscle lineage through expression of these genes.

Many of the premyogenic mesoderm genes regulate each other’s expression and eventually drive commitment to the skeletal muscle lineage. In P19 cells, Meox1 and Gli2 overexpression activated each other’s expression and dominant negative mutants of Meox1/EnR or Gli2/EnR downregulated Pax3 and inhibited skeletal myogenesis [26]. In similar studies, Pax3 overexpression induced myogenesis by upregulating Meox1, Six1, and Eya2 and dominant negative Pax3/EnR reduced expression of these factors, inhibiting myogenesis [27]. In turn, Six1 and Eya2 act synergistically to regulate the expression of Pax3 during myogenesis in the embryo [24], [28]. These results suggest the presence of positive regulatory loops between Pax3, Gli2, Meox1, Six1, and Eya2 during skeletal myogenesis [24], [26], [28].

Foxc1 expression regulates the expression of Pax7 in chick intermediate mesoderm [29]. In P19 cells, β-catenin, Gli2, and Meox1 regulated the expression of Foxc1/2, while Foxc1 overexpression upregulated Pax3, although these cells did not continue to differentiate [14]. Another factor shown to be involved with skeletal myogenesis is Sox7, which can determine the fate of mesodermal derivatives by regulating the expression of mesoderm-inducing genes in *Xenopus* [30]. In contrast to Foxc1, Sox7 overexpression upregulated Pax3/7, Meox1, and Foxc1, leading to enhanced skeletal myogenesis [31].

The details and complexity of how these positive regulatory loops interact with each other during the formation of myogenic progenitor cells still remains unclear. However, it is known that these genes regulate the commitment of skeletal muscle progenitors to skeletal muscle by activating the expression of the myogenic regulatory factors (MRFs), Myf5, MyoD, Myogenin and MRF4 [32]. The MRFs are basic helix loop helix (bHLH) transcription factors that regulate terminal differentiation into skeletal myocytes by driving muscle-specific gene expression. MRFs heterodimerize with ubiquitous E-protein bHLH transcription factors, and together they bind to E-box (CANNTG) consensus sites upstream of muscle-specific genes [33], [34], [35].

The Vitamin A derivative, all-trans retinoic acid (RA), is a morphogen that plays an important role during skeletal myogenesis and is formed through the oxidation of all-trans retinal by the enzyme retinal dehydrogenase (RALDH) (reviewed in [36]). RA induces cellular responses by modulating gene expression through interactions with retinoic acid receptors (RARs) that heterodimerize with retinoid X receptors (RXRs) which together bind retinoic acid response elements (RARE). Exposure of P19, mES, and hES cells to RA concentrations between 3 and 30 nM enhances the formation of skeletal muscle, predominantly by stimulating premyogenic mesoderm gene expression [10], [12], [37]. Furthermore, RA could enhance β-catenin transcriptional activity in aggregated P19 cells but could not bypass the inhibition of skeletal myogenesis by β-cat/EnR, although Gli2 and Pax3 were upregulated [12].

In the analysis of P19[β-cat/EnR] cells, it is possible that other compensatory pathways may be blocked, since the chromatin in these areas could be condensed and silenced from the recruitment of HDACs by the En-2 repressor domain [38]. Therefore, to determine if β-catenin is essential for skeletal myogenesis and cannot be compensated by the presence of other factors, P19 cell lines expressing short hairpin RNAs (shRNA) targeting β-catenin, termed P19[shβ-cat], were created and these cells were differentiated with or without RA. P19[shβ-cat] cells were deficient in skeletal myogenesis and displayed reduced skeletal muscle precursor gene expression. Furthermore, RA treatment was able to recover or stimulate the expression of many skeletal muscle precursor genes, but could not recover MRF expression or terminal skeletal myogenesis.

## Materials and Methods

### Plasmid Constructs

The Super8XTOPFLASH and the Super8XFOPFLASH reporter constructs were the generous gift of the Moon lab (University of Washington). These constructs contain a promoter with 8 copies of the TCF/LEF binding sites driving expression of the firefly luciferase gene. The Super8XFOPFLASH construct contains mutated TCF/LEF binding sites, rendering them inactive.

DNA constructs encoding short hairpin RNAs (shRNAs) complementary to two regions within the mRNA of mouse β-catenin (NM_007614) were created using annealed oligonucleotides encoding sequences from 3056 bp to 3076 bp (5′-TTTGGTTATCAAACCCTAGCCTTCTCAAGAGAAAGGCTAGGGTTTGATAACGCTTTTTT-3′ and 5′-CTAGAAAAAAGCGTTATCAAACCCTAGCCTTTCTCTTGAGAAG GCTAGGGTTTGATAAC-3′) or from 312 bp to 334 bp (5′-TTTGAATCCATTCTG GTGCCACCTTCAAGAGAGGTGGCACCAGAATGGATTCCTTTTTT-3′ and 5′-CT AGAAAAAAGGAATCCATTCTGGTGCCACCTCTCTTGAAGGTGGCACCAGAATGGATT-3′). The annealed oligonucleotides were cloned into the BbsI and XbaI restriction sites of the mU6pro vector, a generous gift from David Turner (University of Michigan, Ann Harbor, MI) [39]. One control plasmid, created using scrambled sequences has been described [31]. Another control plasmid was created with primers 5′-TTTGACAAGATGAAGAGCACCAATTCAAGAGATTGGTG CTCTTCATCTTGTTGTTTTTT -3′ and 5′- CTAGAAAAAACAACAAGATGAAGAGCA CCAATCTCTTGAATTGGTGCTCTTCATCTTGT -3′.

### Cell Culture and Differentiation

The P19 embryonal carcinoma cell lines were propagated in culture by plating 450,000 cells into 100 mm tissue culture plates (Corning Incorporated, Corning, NY) with α-minimum essential medium (MEM) (Gibco, Grand Island, NY) containing 10% fetal bovine serum (PAA, Etobicoke, ON) [11]. The cells were grown for 2 days at 37°C and 5% CO_2_ until they were confluent and then harvested for analysis or re-plated for maintenance or differentiation.

The P19 cell lines were differentiated following a 9 day protocol [11]. On day 0, 450,000 cells were plated into 100 mm petri dishes (Fisher Scientific Company, Ottawa, ON), allowing for the formation of cellular aggregates. Aggregates were maintained until day 4, after which they were plated into tissue culture dishes, for harvesting RNA on day 5 and day 9 respectively. During the 4-day aggregation phase only, the media contained 1% dimethyl sulfoxide (DMSO) in the presence or absence of 3 nM RA. Differentiations were also performed where P19 cells were aggregated for 4 days in the presence and absence of 20 mM LiCl.

P19 cells were transfected with short hairpin constructs by using the FuGene transfection reagent (Roche Applied Sciences, Indianapolis, IN). The cells were co-transfected with either 0.8 µg of the shβ-catenin or shControl constructs and with 0.8 µg of a plasmid conferring puromycin resistance. Colonies were selected with puromycin for 10 days and subsequently pooled, termed P19[shβ-cat] cells or P19[shControl] cells. Experiments shown represent results from 2 independent pooled populations generated from 2 different shRNA sequences targeting β-catenin, repeated 2 or 3 times.

### Promoter Analysis

P19 cell lines were transiently transfected at ∼50–60% confluency in monolayer in 60 mm tissue culture dishes (Corning, Corning, NY) with 1.0 µg of Renilla luciferase and either Super8XTOPFLASH or the Super8XFOPFLASH reporter constructs using the Fugene transfection reagent (Roche Applied Sciences, Indianapolis, IN). The following day, the cells were aggregated and treated with or without 20 mM LiCl in 60 mm petri dishes coated with 1% agarose. The cells were harvested two days after transfection using the Dual Luciferase Kit (Promega, Madison, WI) and luciferase activity was measured using a GLOMAX 96 microplate luminometer with dual injectors (Promega, Madison, WI). Luciferase activity was normalized to Renilla activity and represented as fold changes over Super8XFOPFLASH transfected control cell luciferase activity.

### Immunofluorescence

On day 9, P19 cultures growing on gelatin-coated coverslips were fixed by treatment with methanol. Fixed cells were incubated overnight with MF20 monoclonal antibodies against the muscle-specific marker, Myosin Heavy chain (MHC) as described [40] and with anti-desmin antibodies (mab3430, Millipore, Billerica, MA). Subsequently, Cy3-linked goat anti-mouse IgG2b and Alexa Fluor488 goat anti-mouse IgG1 secondary antibodies (Jackson ImmunoResearch, West Grove, PA) were applied to the cells at a 1∶100 and 1∶200 dilution with PBS, respectively, for 1 hour. Cells were visualized with a Leica DMI6000 B microscope (Leica Microsystems Inc., Richmond Hill, ON) and pictures were acquired using a Micropublisher 3.3 RTV camera (Q Imaging, Surrey, BC). Myogenic differentiation was quantified by performing either manual or automated cell counts using the Volocity software (PerkinElmer Inc., Waltham, MA) and represented as a proportion of total nuclei.

### Reverse Transcription and Quantitative Real Time Polymerase Chain Reaction (QPCR)

RNA was isolated from cells using the RNeasy kit (Qiagen, Maryland, USA) on days 0, 5 and 9 of the differentiation. 1 µg of RNA from each sample was reverse transcribed using the Quantitect Reverse Transcription kit (Qiagen, Maryland, USA). QPCR was carried out using either a Mastercycler realplex or ABI 7300 machine (Eppendorf, Mississaga, ON) and analyzed with either the Realplex software (Eppendorf, Mississaga, ON) or using the SDS software (Applied Biosystems, Streetsville, ON, Canada). For real-time detection of mRNA expression, 1/20 or 1/40 of the total first strand synthesis product as a template for PCR amplification using either the FastStart SYBR Green with ROX (Roche Applied Sciences, Laval, Québec) or the GoTaq QPCR Master Mix (Promega corporation, Madison, WI) respectively. Each reaction was carried out in duplicate as described [40]. The gene primers utilized have been described previously [31], [40], [41]. Fold changes were calculated using the comparative Ct method as described earlier [42]. The resulting Ct values were normalized to either β-actin or GAPDH.

### Western Blot Analysis of Nuclear Extracts

P19[shControl] and P19[shβ-cat] cells were differentiated with LiCl or DMSO as described above and harvested on days 1 or 5, respectively, by scraping cells in sterile PBS. Cells were collected and pellets were resuspended in 3 ml of ice-cold Buffer B (20 mM Tris pH 7.4, 10 mM KCl, 3 mM MgCl_2_, 0.1% NP40, 10% glycerol) with a 25G 1 ½ needle (BD Transduction) and incubated 10 min on ice. The lysed cells were centrifuged at 1430 g for 10 min at 4°C. The supernatant was collected as the cytoplasmic fraction. The remaining nuclear pellet was resuspended in 2.25 ml of S1 buffer (final concentration of 0.25 M Sucrose, 10 mM MgCl_2_) and was layered over a 2.25 ml cushion of S3 buffer (0.88 M Sucrose, 0.5 mM MgCl_2_). Cellular fractions were solubilized in RIPA buffer (50 mM Tris pH 7.5, 150 mM NaCl, 1% NP-40, 0.5% sodium deoxycholate). Nuclear lysate was sonicated on ice using a microtip with 21×10 s pulses at 25% amplitude with a 10 s pause in between each pulse (Vibra-cell Sonics & Materials). The lysates were then clarified by centrifugation at 2800 g for 10 min at 4°C. Protein concentrations were determined using a standard Bradford assay (Biorad). Nuclear protein (15 µg) was diluted to a final concentration of 1× LDS buffer (Invitrogen) and separated on a 4–12% NuPAGE Bis-Tris Precast Gel. Resolved proteins were transferred to a nitrocellulose membrane, which was blocked with 5% Milk in 0.5% TBST and reacted with antibodies against mouse β-catenin (Sigma) or mouse RNA-polII (clone CTD4H8; Millipore, Billerica, MA ), at a dilution of 1∶2000 or 1∶1000, respectively. Chemiluminescence was generated using appropriate secondary horseradish peroxidase-conjugated antibodies (Thermoscientific), followed by incubation with the Luminata™ Crescendo substrate (Millipore, Billerica, MA) and detected and quantified using the Fuji LAS 4000 mini imaging system and Multigauge quantitation software.

Successful isolation of cytoplasmic and nuclear protein fractions was confirmed by α-tubulin and RNA polymerase II immunoblots. The nuclear fractions were found to contain <20% of cytoplasmic protein (data not shown).

### Chromatin Immunoprecipitation (ChIP)

P19 cells were differentiated for two days in the presence of DMSO or DMSO+RA and processed according to methods described previously [31]. Immunoprecipitation was performed with either 5 µg of β-catenin antibody (clone 15B8, Sigma-Aldrich) or 5 µg of IgG antiserum (Zymed Laboratories, California). Samples were treated with 20 µg RNase A and 40 µg Proteinase K and DNA purified using Qiagen’s PCR purification kit (Qiagen, Mississauga, ON). Enrichment of binding sites was analyzed using SYBR Green real-time PCR, as described above. Primer sequences have been described previously and were tested for equivalent efficiency [31].

### Statistical Analysis

The Student’s t-test or one-way ANOVA’s were used to calculate the statistical significance of differences between means, where a *p-value ≤0.05 was considered significant.

## Results

### P19[shβ-cat] Cells Exhibit Reduced β-catenin Transcriptional Activity

**Figure 1:**
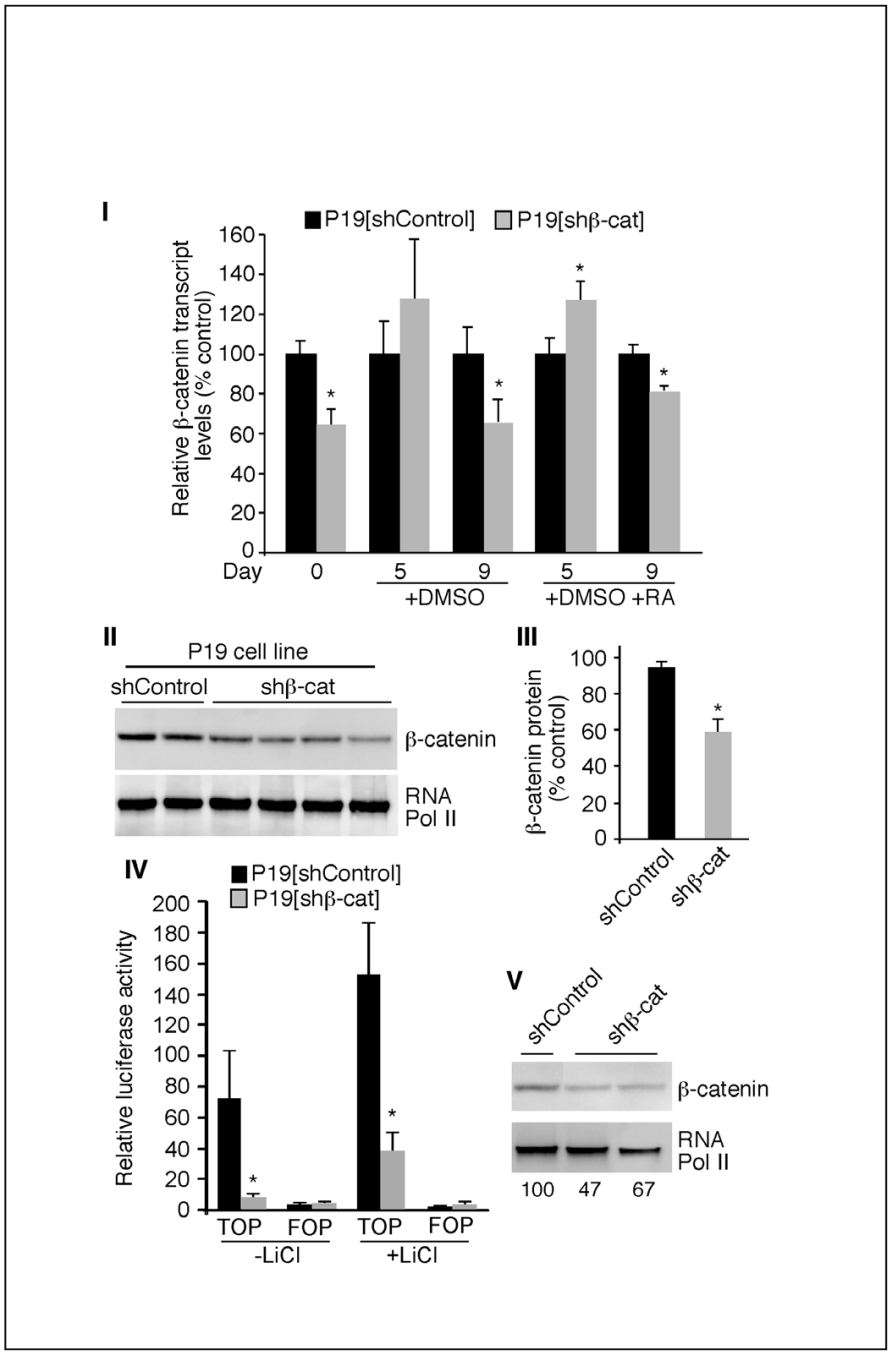
β-catenin transcriptional activity and protein levels were reduced in P19[shβ-cat] cells. *Panel I:* P19[shControl] and P19[shβ-cat] cells were differentiated in the presence of 1% DMSO with or without 3 nM RA. β-catenin transcript levels were determined by QPCR, which was performed from RNA harvested on the days indicated (n = 12 for day 0, n = 5 for day 5, n = 4 for day 9). Changes in gene expression were normalized to β-actin and expressed as a percentage of P19[shControl] cells under each condition. Error bars represent ± SEM. Student’s t-test was used to assess statistical significance, with *p≤0.05. *Panel II&III*: β-catenin protein was detected by western blot analysis using protein isolated from the nuclei of P19[shControl] and P19[shβ-cat] cells differentiated for five days with DMSO. Relative β-catenin protein levels were quantified by densitometry and normalized to RNA polymerase II protein levels (loading control). Error bars represent ± SEM (n = 4). Panel IV: P19[shβ-cat] and P19[shControl] cells were aggregated for 1 day in the presence or absence of 20 mM LiCl. Firefly luciferase activity was measured from cells co-transfected with either Super8XTOPFLASH (TOP) or Super8XFOPFLASH (FOP) and the Renilla control construct. All firefly luciferase activity was normalized to Renilla and represented as the fold change over P19[shControl] FOP luciferase activity. Error bars correspond to the average ± SEM (n = 4). The Student’s t-test was used to assess statistical significance, where *p-value ≤0.05 was considered significant. Panel V: β-catenin protein was detected by western blot analysis using protein isolated from the nuclei of P19[shControl] and P19[shβ-cat] cells differentiated for one day with LiCl, as described in Panel II. Numbers represent quantification by densitometry of β-catenin protein levels, normalized to RNA Pol II.

P19[shβ-cat] cell lines were created to determine if β-catenin expression was essential for efficient skeletal myogenesis in P19 cells. The efficacy of downregulation of β-catenin mRNA, protein, and transcriptional activity was determined in P19[shβ-cat] cells compared to P19[shControl] cells, using QPCR, western blot analysis of nuclear extracts, and luciferase activity, respectively (Fig. 1). P19[shβ-cat] and P19[shControl] cells were differentiated by cellular aggregation with DMSO in the presence and absence of RA and total RNA and was harvested for examination by QPCR (Fig. 1, *Panel I*). β-catenin transcript levels were significantly reduced on days 0 and 9, with and without RA treatment, in P19[shβ-cat] cells when compared to P19[shControl] cells (Fig. 1, *Panel I*). The expression of β-catenin transcripts on day 5 was not downregulated in P19[shβ-cat] cells treated with DMSO and was slightly upregulated with DMSO and RA. Thus, β-catenin transcript levels were reduced in P19[shβ-cat] cells at the beginning and end of the differentiation time course under both treatment conditions.

To determine whether the β-catenin mRNA levels correlated with β-catenin protein, western blot analysis was performed on nuclear extracts from cells differentiated for 5 days with DMSO during the aggregation phase, and quantified (Fig. 1, *Panels II and III*). The abundance of nuclear β-catenin protein was significantly reduced, by approximately 40%, in P19[shβ-cat] cells compared to P19[shControl] cells (Fig. 1 *Panel III*). To test whether the knockdown had a significant effect on β-catenin transcriptional activity, luciferase assays were performed. P19[shβ-cat] and P19[shControl] cells were transiently transfected with either the Wnt-responsive reporter construct Super8XTOPFLASH or the mutant Wnt-nonresponsive reporter construct Super8XFOPFLASH and were aggregated for one day with or without LiCl, a known inducer of Wnt signalling [43] (Fig. 1, Panel IV). The β-catenin activity of P19[shβ-cat] cells was greatly reduced compared to P19[shControl] cells after aggregation. Further, while LiCl induced a large increase in β-catenin transcriptional activity in P19[shControl] cells, only a smaller increase was observed in P19[shβ-cat] cells (Fig. 1, Panel IV). Western blot analysis of nuclear protein extracts from these cultures showed approximately a 40% reduction in nuclear β-catenin protein levels (Fig. 1, Panel V). Thus, P19[shβ-cat] cells are deficient in β-catenin transcriptional activity and have significantly lower levels of β-catenin mRNA and nuclear protein.

### Loss of β-catenin Activity Inhibits Skeletal Myogenesis

**Figure 2:**
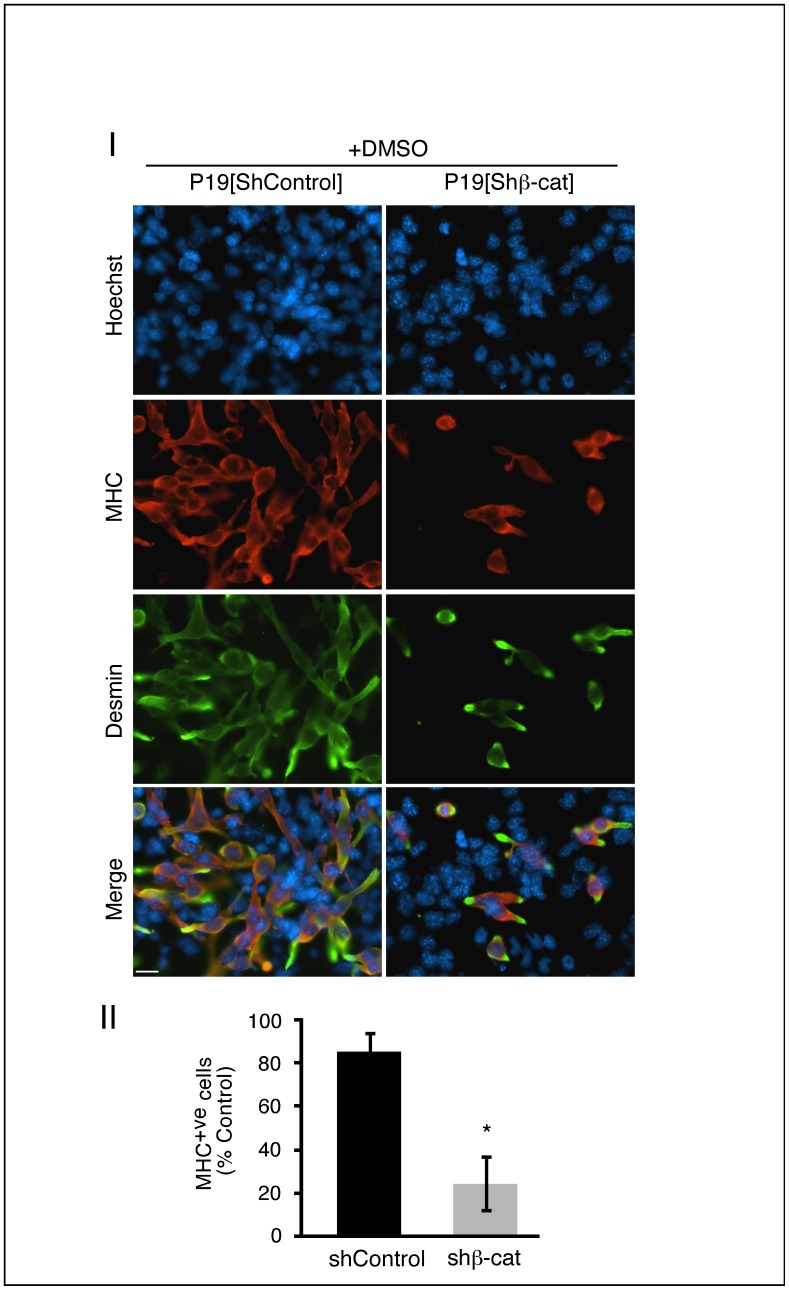
Skeletal myogenesis was reduced in P19[Shβ-cat] cells. Aggregated P19[shControl] and P19[shβ-cat] cells were differentiated in the presence of 1% DMSO. Panel I: On day 9 of the differentiation, cells were fixed for immunofluorescence analysis using anti-Myosin heavy chain monoclonal antibodies (MHC; red) or anti-desmin antibodies (green), to visualize muscle cells and Hoechst dye to visualize cell nuclei (bar = 20 µm). Panel II: The degree of skeletal myogenesis was quantified by counting the number of MHC^+ve^ cells and expressed as the percentage of MHC^+ve^ cells in P19[shControl] cells. Error bars represent ± SEM (n = 6; 9000–11000 cells counted/condition). The Student’s t-test was used to assess statistical significance, where *p-value ≤0.05.

Since P19[shβ-cat] cells were shown to have highly reduced β-catenin nuclear activity, we set out to determine how this affected *in vitro* skeletal myogenesis. To assess the degree of skeletal muscle development, immunofluorescence was performed on day 9 cultures with an antibody against myosin heavy chain (MHC; red), termed MF20, and the percentage of total cells expressing MHC was calculated (Fig. 2 *Panels I and II*). Cultures were co-stained with an antibody to desmin (green). Since the structural striated muscle genes are present in both developing cardiac and skeletal muscle, MHC-positive cells were identified as skeletal muscle by their bipolar morphology, which contrasts the rounded shape of cardiomyocytes [12]. When P19[shβ-cat] cells were differentiated with DMSO, there was an ∼80% reduction in skeletal myogenesis compared to P19[shControl] cells, respectively (Fig. 2). Therefore, β-catenin is essential for efficient skeletal myogenesis, supporting and extending our previous findings with dominant negative β-catenin overexpression [12].

**Figure 3:**
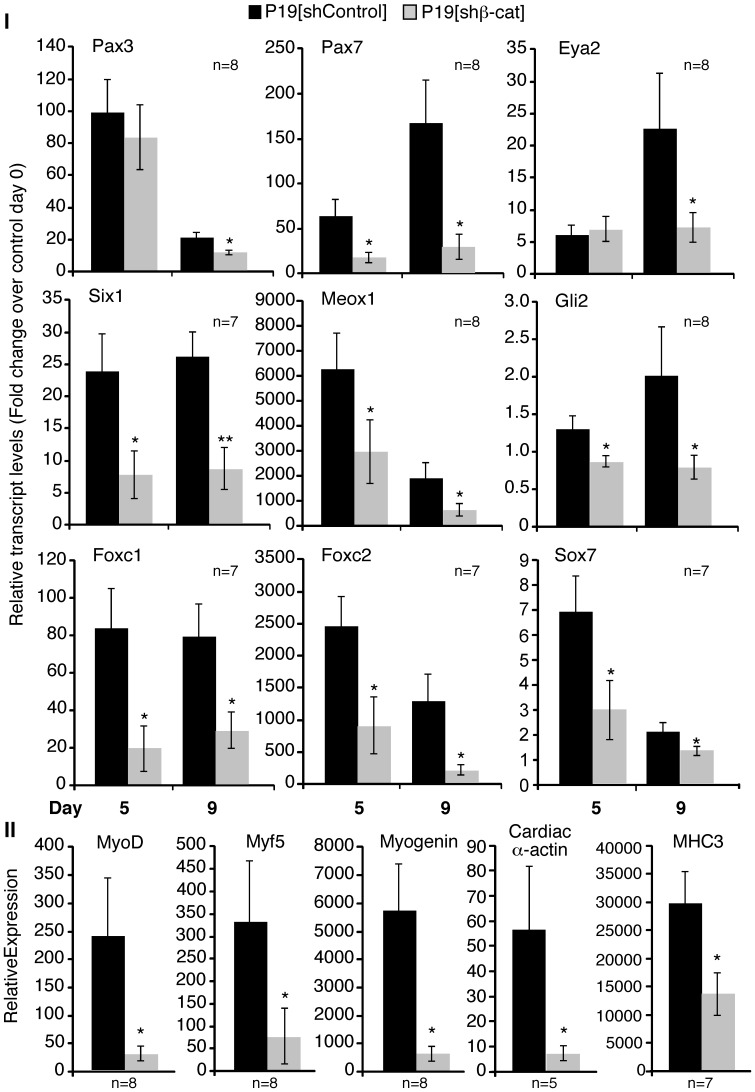
Skeletal muscle, myoblast, and muscle precursor gene expression was reduced in P19[Shβ-cat] cells. Aggregated P19[shControl] and P19[shβ-cat] cells were differentiated in the presence of 1% DMSO. RNA was harvested on days 0, 5 and 9 of the differentiation. QPCR was performed to quantify the transcript levels of the indicated genes. Changes in gene expression were normalized against β-actin and represented as fold change over day 0 transcript levels in P19[shControl] cells. Error bars correspond to the average ± SEM. The Student’s t-test was used to assess statistical significance, where *p-value ≤0.05.

To decipher the stage at which skeletal myogenesis was inhibited, we examined changes in gene expression by QPCR analysis during myogenic differentiation of P19[shβ-cat] cells. On day 5 of differentiation, expression of genes representing the somitic/premyogenic mesoderm stages, such as Meox1, Gli2, Pax3, Six1, Foxc1 and Foxc2 were examined and, on day 9 of differentiation, genes representing the myoblast stage, such as MyoD, myogenin, and Myf-5 were examined [11], [14], [40](summarized in Table 1). P19[shβ-cat] cells differentiated in the presence of DMSO alone displayed significant down regulation of all the somitic/premyogenic mesoderm genes, including Pax3/7, Eya1, Six1, Meox1, Gli2, Foxc1/2, and Sox7, on day 5 and/or day 9 (Fig. 3, Panel I; summarized in Table 2). Transcripts for MRFs and for the structural genes MHC3 and cardiac α-actin, were highly reduced on day 9 in P19[shβ-cat] cells when compared to P19[shControl] cells (Fig. 3, Panel II; summarized in Table 2). MyoD, Myf5, Myogenin, and MHC3 expression was significantly downregulated by 80–90%. (Fig. 3, *Panel II*), whereas MHC3 was downregulated by ∼55%. Therefore, β-catenin was required for efficient *in vitro* skeletal myogenesis as early as the skeletal muscle progenitor stage, since the loss of β-catenin resulted in a downregulation of all skeletal muscle progenitor genes tested.

**Table 1:** Summary of changes in gene expression in P19 cells treated with DMSO with or without 3 nM RA, compared to untreated cells.

		P19 cells	
Days: Markers	Genes	DMSO	DMSO & RA
Day 5: Somitic/premyogenic mesoderm	Pax3	+	++
	Pax7	+	++
	Eya2	+	++
	Six1	+	++
	Meox1	+	++
	Gli2	+	+
	Foxc1	+	+
	Foxc2	+	+
	Sox7	+	+
Day 9: Myoblast/Myocyte	MyoD	+	++
	Myf5	+	++
	Myogenin	+	++
	Cardiac α-actin	+	++
	MHC	+	++

Changes in gene expression are indicated as follows: − = decrease;+ = increase;++ = greater increase (Fig. 3, Fig. 5, [12]).

**Table 2:** Summary of changes in gene expression in P19[shβ-cat] cultures compared to same-day P19[shControl] cultures after differentiation in DMSO, with and without RA (− = decrease;+ = increase; NC = No change; NE = not expressed; ND = not determined; * = Enhanced or recovered gene expression due to RA treatment)(Data from Figs. 3 and 5).

		P19[shβ-cat] cultures			
		Day 5		Day 9	
Markers	Genes	DMSO	DMSO & RA	DMSO	DMSO & RA
Somitic/premyogenic mesoderm	Pax3	NC	NC	−	+ *
	Pax7	−	+ *	−	NC *
	Eya2	NC	−	−	NC *
	Six1	−	−	−	+ *
	Meox1	−	−	−	+ *
	Gli2	−	+ *	−	−
	Foxc1	−	−	−	+ *
	Foxc2	−	−	−	−
	Sox7	−	NC *	−	−
Myoblast/Myocyte	MyoD	NE	NE	−	−
	Myf5	ND	ND	−	−
	Myogenin	NE	NE	−	−
	Cardiac α-actin	NE	NE	−	−
	MHC3	NE	NE	−	−

### β-catenin Associates with Regulatory Regions of the Sox7 Gene

**Figure 4:**
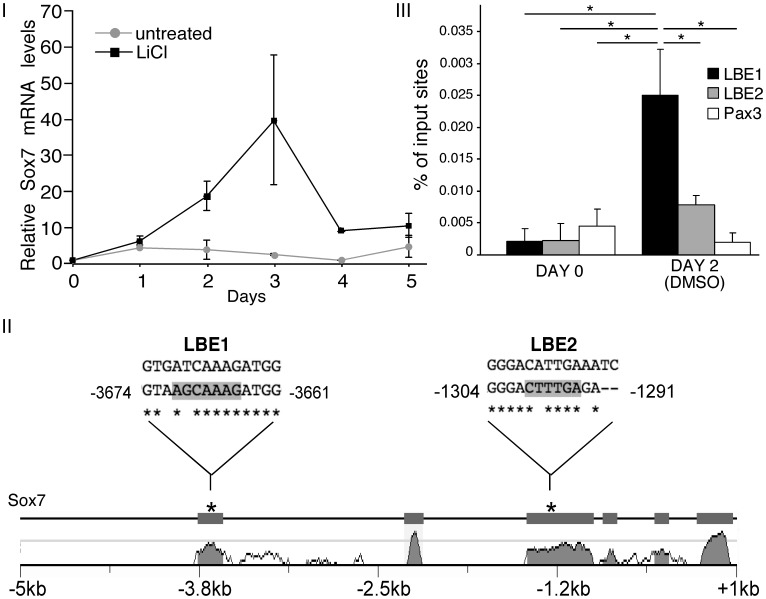
Sox7 expression is directly modulated by Canonical Wnt signalling. *Panel I:* P19 cells were differentiated in the presence or absence of 20 mM LiCl for 5 days. Changes in gene expression were analyzed by QPCR, normalized against GAPDH and represented as the average fold change over day 0 expression levels. *Panel II:* Conserved LEF/TCF binding elements (LBE) were identified by aligning DNA sequences from the mouse and human genomes using MULAN [44]. *Panel III:* Crossed-linked chromatin was isolated from P19 cells on days 0 and 2 of DMSO-induced differentiation. ChIP was performed using an anti-β-catenin antibody, analyzed by QPCR with the indicated primers, and represented as the average percent of input sites that were immunoprecipitated. Error bars correspond to the ± SEM (n = 4). A one-way ANOVA was used to assess the statistical significance, where *p-value ≤0.05.

Previously we had shown that Gli2, Meox1, Pax3, Six1 and Foxc1/2 could be upregulated by expression of a constitutively activated β-catenin [13], [14] and that Foxc1/2 could be upregulated by treatment of aggregated P19 cells with LiCl [14]. Since Sox7 induces skeletal myogenesis in P19 cells, we set out to determine if it could also be upregulated by LiCl treatment. P19 cells were aggregated with and without 20 mM LiCl and examined for Sox7 expression. While cells aggregated in the absence of LiCl did not upregulate Sox7 to any appreciable levels, cells aggregated in the presence of LiCl upregulated Sox7 40-fold over control cells (Fig. 4, Panel I). Thus, canonical Wnt signalling regulates Sox7 expression either directly or indirectly.

Since all of the tested skeletal muscle progenitor genes were regulated by the gain or loss of β-catenin, we were interested in knowing if this relationship was direct or indirect. Previous studies have shown that β-catenin associates with the regulatory regions of *Foxc1*, although Foxc1 overexpression could not initiate the pathway of skeletal myogenesis in P19 cells [14]. To determine if β-catenin could bind to the regulatory regions of genes that can induce skeletal myogenesis in P19 cells, the promoter regions of *Sox7* and *Pax3* were examined via chromatin immunoprecipitation experiments (ChIP). The 5 kb regions upstream of *Pax3* and *Sox7* were examined for conserved LEF/TCF binding sites using MuLAN software as described [44]. Two conserved sites (LBE1 and LBE2) were identified in the *Sox7* upstream region (Fig. 4, Panel II) and one conserved LEF/TCF site was identified for *Pax3*.

To perform the ChIP-QPCR, chromatin was isolated from P19 cells grown in monolayer or after mesoderm induction following aggregation with DMSO for two days. Subsequently, chromatin was extracted and immunoprecipitated with an antibody against β-catenin, followed by QPCR using primer pairs that flank LBE1, LBE2, or the conserved LEF site in the *Pax3* promoter region. The ChIP-QPCR indicated that 0.025% of input sites of day 2 cells treated with DMSO were associated with β-catenin at the LBE1 site, compared to 0.008% at the LBE2 site and 0.002% at the *Pax3* promoter (Fig. 4, *Panel III*). The enrichment of immunoprecipitated sites at the LBE1 region on day 2 was statistically significant when compared to the enrichment at LBE2 and *Pax3*, as well as to the enrichment identified on day 0 for all the loci. Thus, β-catenin is associated with a *Sox7* gene regulatory region in a population of aggregated P19 cells.

### RA Enhances the Expression of Several Skeletal Muscle Precursor Genes, but not MRF Expression in P19[shβ-cat] Cells

**Figure 5:**
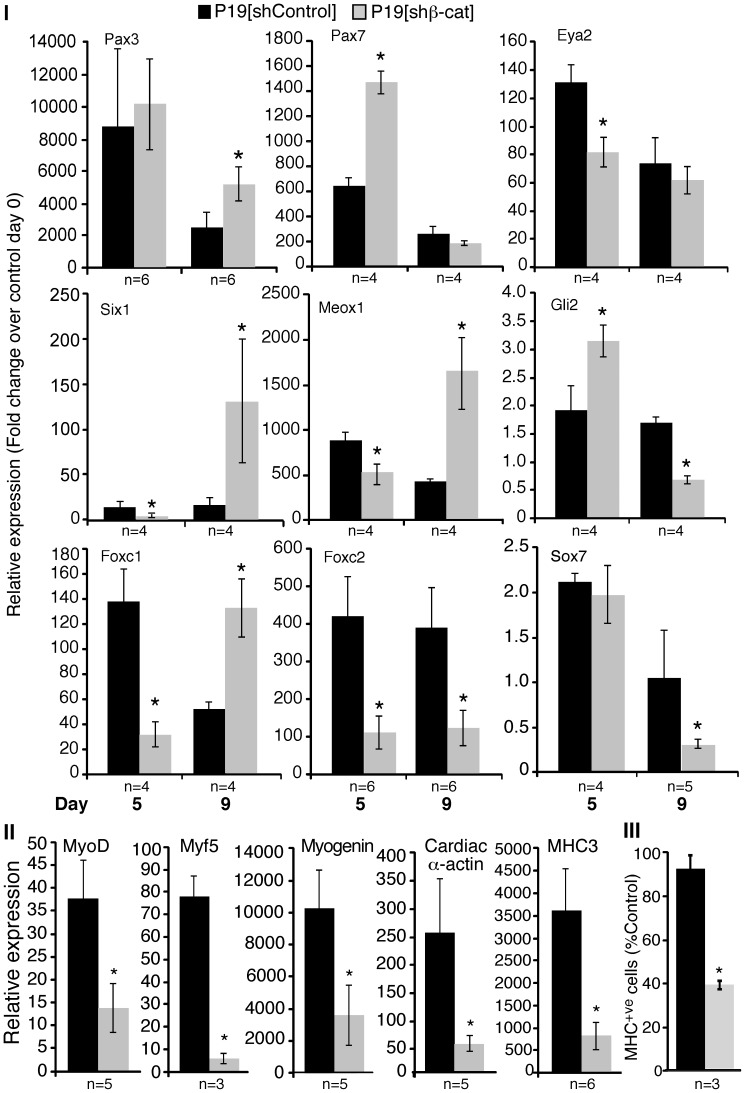
RA can recover the expression of most skeletal muscle precursor genes in P19[shβ-cat] cells. Aggregated P19[shControl] and P19[shβ-cat] cells were differentiated in the presence of 1% DMSO with 3 nM RA. *Panels I & II:* RNA was harvested on days 0, 5 and 9 of the differentiation and analyzed by QPCR for all days (Panel I) or days 0 & 9 (Panel II). Changes in gene expression were normalized against β-actin represent fold change over day 0 levels in P19[shControl] cells. Error bars correspond to the ± SEM. Panel III: Cultures were fixed on day 9 for immunofluorescence with an anti-MHC antibody. The number of MHC^+ve^ cells were counted and expressed as a percentage of the control cells. Error bars represent ± SEM (n = 6). The Student’s t-test was used to assess statistical significance, where *p-value ≤0.05.

Previously we had shown that RA could not bypass the inhibition of skeletal myogenesis by a dominant negative β-catenin/engrailed fusion protein [12]. While Pax3/7 expression was upregulated by RA treatment of P19[β-cat/EnR] cells, Meox1 and MyoD were not [12]. To determine if the lack of compensation was due to the use of a dominant negative engrailed fusion protein, P19[shβ-cat] cells were examined for their ability to differentiate in the presence of RA. Gene expression was measured using QPCR analysis, with RNA harvested from day 0, 5 and 9 cultures and RA was found to enhance MRF expression and myogenesis in control P19 cultures (Fig. S1), in agreement with previous reports [12]. During RA-induced differentiation, the expression of several skeletal muscle precursor genes were recovered or enhanced in P19[shβ-cat] cells (Fig. 5, Panel I; summarized in Table 2). Specifically, Pax3/7, Eya2, Six1, Meox1, and Foxc1 transcript levels were recovered or increased on day 9 when compared to P19[shControl] cells. Gli2 and Sox7 expression was upregulated and recovered, respectively, only on day 5. Pax7 expression on day 5 was also upregulated, and the expression on day 9 was recovered. Notably, Eya2 expression became significantly downregulated in the presence of RA on day 5, but by day 9 its expression was comparable to P19[shControl] cells. Lastly, Foxc2 expression was still reduced on both days in the presence of RA. Thus, RA is sufficient to recover or upregulate expression of many premyogenic mesoderm genes, on day 5 and/or day 9 of differentiation, even when β-catenin transcriptional activity is reduced.

Since RA is able to recover, at least partially, the expression of several skeletal muscle progenitor genes in P19[shβ-cat] cells, we sought to determine if there was recovery or enhanced expression of myoblast or terminal differentiation markers. As shown by QPCR analysis on day 9 of differentiation, MRF transcript levels and terminal differentiation markers were still reduced in P19[shβ-cat] cells when compared to P19[shControl] cells during RA-induced differentiation (Fig. 5, Panel II). Consistent with the reduction in MRF and muscle differentiation markers, quantification of the number of MHC-positive skeletal myocytes by immunofluorescence revealed a reduction in overall skeletal myogenesis (Fig. 5, Panel III). Thus, reduced MRF and terminal differentiation marker expression during RA-induced differentiation indicates the inability of RA to recover the expression of genes further downstream of premyogenic mesoderm.

## Discussion

P19[shβ-cat] cells were deficient in undergoing skeletal myogenesis after cellular aggregation in the presence of DMSO. Downregulated expression was observed for premyogenic mesoderm genes, including Pax3/7, Six1, Eya2, Meox1, Gli2, Foxc1/2, and Sox7, and the myoblast markers, MyoD, Myf5 and myogenin. Sox7 was identified as a potential downstream target of Wnt signalling, because it was upregulated by LiCl treatment of aggregated P19 cells and β-catenin was associated with conserved sites in the Sox7 promoter, but not the Pax3 promoter, by ChIP-QPCR analysis. RA was unable to enhance skeletal myogenesis in P19[shβ-cat] cells, although RA upregulated or recovered the expression of most skeletal muscle precursor genes on day 5 and/or day 9, including Pax3/7, Eya2, Six1, Meox1, Gli2, Foxc1, and Sox7. However, the expression of Sox7, Gli2, MRFs and terminal differentiation markers was not recovered under these conditions on day 9. These findings are consistent with a model in which both Wnt and RA signalling are important for regulating skeletal muscle precursor genes.

### β-catenin mRNA and Protein Levels did not Always Correlate with Reduction in β-catenin Activity

P19[shβ-cat] cells showed a significant reduction in nuclear β-catenin protein levels on days 1 and 5 of differentiation, and a dramatic loss in the ability of P19[shβ-cat] cells to activate a Wnt-reporter and differentiate into muscle. However, we did not always observe a consistent correlation between β-catenin transcript levels, protein levels, and nuclear activity. For example, while the maximum loss of mRNA was about 40% on day 0, correlating with a 40% loss of β-catenin protein on day 5 of differentiation, this did not correlate with the lack of mRNA downregulation on day 5. Furthermore, the >75% loss of nuclear β-catenin activity was greater than the observed loss of about 40% β-catenin protein levels on day 1 of differentiation. β-catenin is a multifunctional protein, with the majority of protein functioning in cell adhesion [45] and a minority of protein functioning to transduce Wnt signals. It is likely that the complex subcellular distribution of β-catenin, leading to different levels of protein stability, augmented the difficulties of observing true changes in nuclear β-catenin protein levels. Furthermore, in our nuclear extract protocol, there was still up to 20% of cytoplasmic contamination in some experiments, that could mask the downregulation of nuclear β-catenin (data not shown). However, the dramatic loss of the ability of β-catenin to activate a reporter gene confirms the validity of the P19[shβ-cat] cell line.

Consistent with our current observations, we previously showed that very small changes in total β–catenin mRNA levels can create large changes in β-catenin activity when we examined P19 cells overexpressing an activated form of β-catenin, termed P19[β-catenin*] cells [13]. Only minor differences in total protein levels were observed between P19[control] cells and P19[β-catenin*] cells when examined by immunofluorescence with an anti-β-catenin antibody. However, when examined with an anti-HA antibody, the expression of exogenous β-catenin was clearly present [13]. Therefore, for gain- and loss-of function experiments for β-catenin, nuclear β-catenin is a much better indication of function than total protein, but may still overestimate nuclear β-catenin activity.

### Knockdown of β-catenin Nuclear Activity Inhibits Skeletal Myogenesis

Previously, it was shown that functional β-catenin is necessary for skeletal myogenesis in P19 cells through the expression of β-cat/EnR [12], [13]. In these cells, skeletal myogenesis was inhibited and the expression of several skeletal muscle precursor genes, including Pax3/7, Six1, Gli2, and Meox1 was abolished. In this study, knockdown of β-catenin’s nuclear activity using shRNAs resulted in reduced expression of the skeletal muscle precursor genes shown previously as well as of Eya2, Foxc1 and Foxc2 on day 5 and/or day 9 during DMSO-induced differentiation. Thus, these results extend our previous findings by showing that β-catenin is essential for efficient *in vitro* skeletal myogenesis and cannot be compensated by other endogenous factors.

### RA can Recover or Enhance the Expression of Most Skeletal Muscle Precursor Genes When β-catenin Nuclear Activity is Reduced, but cannot Recover MRF Expression

When P19[shβ-catenin] cells were differentiated with 1% DMSO and 3 nM RA, all of the skeletal muscle precursor genes examined were at least partially recovered or enhanced on day 5 and/or day 9, except for Foxc2. The increases in expression observed for Pax3/7 and Gli2 agree with previous increases shown for RA-treated P19[β-cat/EnR] cells and with a demonstrated direct interaction between RARs and the Pax3 gene regulatory regions [12]. However, P19[β-cat/EnR] cells were not able to upregulate Meox1 in the presence of RA, in contrast to P19[shβ-cat] cells, and despite a demonstrated interaction between RARs and the Meox1 regulatory regions [12]. The lack of RA-induced upregulation of Meox1 in P19[β-cat/EnR] cells compared to P19[shβ-cat] cells indicates that the dominant negative approach prevented compensation of Meox1 expression by RA signalling, justifying the examination of these transcriptional networks using both approaches.

Our findings examined transcription factors not studied previously in P19[β-cat/EnR] cells, showing that Six1, Eya2, Foxc1, and Sox7 expression can also be recovered in P19[shβ-cat] cells by RA signalling. The involvement of Six1 and Eya2 in a positive regulatory loop with Pax3 and Pax7 may partially explain their increased transcription [27], [46]. The RA-stimulated Foxc1 expression agrees with the upregulation by RA of Foxc1 during eye development [47]. We showed that the *Sox7* gene was a potential direct target of β-catenin using ChIP-QPCR. However, we were unable to identify a direct interaction between RARs and the conserved RAREs in the *Sox7* gene (Ryan and Skerjanc, unpublished observations). Reduced Foxc2 expression in both the absence and presence of RA in P19[shβ-catenin] cells suggests that canonical Wnt signalling is essential for its expression and that Foxc2 is not regulated by RA signalling, consistent with its predominant role in regulating vascular smooth muscle development [48]. Overall, the results suggest that the β-cat/EnR dominant negative protein prevented some compensation of gene expression by RA but overall the two approaches gave comparable results.

Although the expression of several skeletal muscle precursor genes are upregulated in the presence of RA, MRF and terminal marker expression was significantly downregulated in P19[shβ-catenin] cells, which agrees with findings in P19[β-cat/EnR] cells [12]. It is possible that skeletal myogenesis is delayed in P19[shβ-cat] cells since RA upregulated the bulk of the premyogenic mesoderm genes only by day 9 [13], [27]. However, the lack of upregulation of Sox7 and Gli2 on day 9 could preclude upregulation of MRFs, as shown in Gli/EnR and Sox/EnR cells [26], [31], as well as in P19 cells with Gli2 knocked-down via shRNA [49]. Thus it is unlikely that the knockdown of β-catenin with RA treatment simply delayed skeletal myogenesis, although it cannot be completely ruled out. It is also possible that MRF expression requires β-catenin nuclear activity, since MyoD function is positively regulated by binding β-catenin, and loss of MyoD function would inhibit the positive regulatory loop required to upregulate and maintain MRF expression [50], [51]. Furthermore, in *Xenopus*, Myf5 expression is highly reduced, when canonical Wnt signalling is inhibited, and TCF/LEF sites have been identified in the early epaxial enhancer of *Myf5,* further suggesting a regulatory role by β-catenin [52].

### Conclusion

In summary, these results demonstrate that the transcriptional activity of β-catenin is required for efficient skeletal muscle precursor gene expression leading to myogenesis in P19 cells. Furthermore, β-catenin can be replaced by RA signalling for at least partial expression of many muscle precursor genes but this did not lead to expression of MRFs or terminal muscle differentiation markers. The enhancement of premyogenic mesoderm genes by RA when nuclear β-catenin is reduced suggests that the RA and Wnt signalling pathways are intricately connected and work together in a complex manner to regulate gene expression during embryonic skeletal muscle development. Insight into the intersection of signalling pathways that regulate key transcriptional networks may lead to novel approaches to treat skeletal muscle diseases.

## Supporting Information

Figure S1 **RA enhanced the expression of MRFs and MHC in P19[shControl] cells.** P19[shControl] cells were differentiated in the presence of 1% DMSO with or without 3 nM RA as described in Materials and Methods. RNA was harvested from day 0 (undifferentiated) and day 9 (differentiated) cells and analyzed using RT-QPCR to quantify the transcript levels of the indicated genes. Data was normalized to β-actin, calculated as fold change relative to day 0 cells and expressed as percent maximum. Error bars indicate +/− SEM. The Student’s t-test was used to assess statistical significance, where **p-value<0.01, n = 3.
(PDF)

## References

[1] Buckingham M, Vincent SD (2009) Distinct and dynamic myogenic populations in the vertebrate embryo. Curr Opin Genet Dev 19: 444–453. 19762225 10.1016/j.gde.2009.08.001

[2] Munsterberg AE, Kitajewski J, Bumcrot DA, McMahon AP, Lassar AB (1995) Combinatorial signaling by Sonic hedgehog and Wnt family members induces myogenic bHLH gene expression in the somite. Genes Dev 9: 2911–2922. 7498788 10.1101/gad.9.23.2911

[3] Galli LM, Willert K, Nusse R, Yablonka-Reuveni Z, Nohno T, et al (2004) A proliferative role for Wnt-3a in chick somites. Dev Biol 269: 489–504. 15110715 10.1016/j.ydbio.2004.01.041

[4] Ikeya M, Takada S (1998) Wnt signaling from the dorsal neural tube is required for the formation of the medial dermomyotome. Development 125: 4969–4976. 9811581 10.1242/dev.125.24.4969

[5] Clevers H (2006) Wnt/β-catenin signaling in development and disease. Cell 127: 469–480. 17081971 10.1016/j.cell.2006.10.018

[6] McBurney MW (1993) P19 embryonal carcinoma cells. Int J Dev Biol 37: 135–140. 8507558

[7] Rossant J, McBurney MW (1982) The developmental potential of a euploid male teratocarcinoma cell line after blastocyst injection. J Embryol Exp Morphol 70: 99–112. 7142904

[8] Vidricaire G, Jardine K, McBurney MW (1994) Expression of the Brachyury gene during mesoderm development in differentiating embryonal carcinoma cell cultures. Development 120: 115–122. 8119120 10.1242/dev.120.1.115

[9] Skerjanc IS (1999) Cardiac and skeletal muscle development in P19 embryonal carcinoma cells. Trends Cardiovasc Med 9: 139–143. 10639728 10.1016/s1050-1738(99)00017-1

[10] Ryan T, Liu J, Chu A, Wang L, Blais A, et al (2012) Retinoic acid enhances skeletal myogenesis in human embryonic stem cells by expanding the premyogenic progenitor population. Stem Cell Rev 8: 482–493. 21735106 10.1007/s12015-011-9284-0

[11] Al Madhoun AS, Mehta V, Li G, Figeys D, Wiper-Bergeron N, et al (2011) Skeletal myosin light chain kinase regulates skeletal myogenesis by phosphorylation of MEF2C. EMBO J 30: 2477–2489. 21556048 10.1038/emboj.2011.153 PMC3116284

[12] Kennedy KA, Porter T, Mehta V, Ryan SD, Price F, et al (2009) Retinoic acid enhances skeletal muscle progenitor formation and bypasses inhibition by bone morphogenetic protein 4 but not dominant negative β-catenin. BMC Biol 7: 67. 19814781 10.1186/1741-7007-7-67 PMC2764571

[13] Petropoulos H, Skerjanc IS (2002) Β-catenin is essential and sufficient for skeletal myogenesis in P19 cells. J Biol Chem 277: 15393–15399. 11856745 10.1074/jbc.M112141200

[14] Savage J, Voronova A, Mehta V, Sendi-Mukasa F, Skerjanc IS (2010) Canonical Wnt signaling regulates Foxc1/2 expression in P19 cells. Differentiation 79: 31–40. 19782461 10.1016/j.diff.2009.08.008

[15] Reijntjes S, Stricker S, Mankoo BS (2007) A comparative analysis of Meox1 and Meox2 in the developing somites and limbs of the chick embryo. Int J Dev Biol 51: 753–759. 17939123 10.1387/ijdb.072332sr

[16] McDermott A, Gustafsson M, Elsam T, Hui CC, Emerson CP Jr, et al (2005) Gli2 and Gli3 have redundant and context-dependent function in skeletal muscle formation. Development 132: 345–357. 15604102 10.1242/dev.01537

[17] Williams BA, Ordahl CP (1994) Pax-3 expression in segmental mesoderm marks early stages in myogenic cell specification. Development 120: 785–796. 7600957 10.1242/dev.120.4.785

[18] Oliver G, Mailhos A, Wehr R, Copeland NG, Jenkins NA, et al (1995) Six3, a murine homologue of the sine oculis gene, demarcates the most anterior border of the developing neural plate and is expressed during eye development. Development 121: 4045–4055. 8575305 10.1242/dev.121.12.4045

[19] Sasaki H, Hogan BL (1993) Differential expression of multiple fork head related genes during gastrulation and axial pattern formation in the mouse embryo. Development 118: 47–59. 8375339 10.1242/dev.118.1.47

[20] Miura N, Wanaka A, Tohyama M, Tanaka K (1993) MFH-1, a new member of the fork head domain family, is expressed in developing mesenchyme. FEBS Lett 326: 171–176. 8325367 10.1016/0014-5793(93)81785-x

[21] Mankoo BS, Skuntz S, Harrigan I, Grigorieva E, Candia A, et al (2003) The concerted action of Meox homeobox genes is required upstream of genetic pathways essential for the formation, patterning and differentiation of somites. Development 130: 4655–4664. 12925591 10.1242/dev.00687

[22] Franz T, Kothary R, Surani MA, Halata Z, Grim M (1993) The Splotch mutation interferes with muscle development in the limbs. Anat Embryol (Berl) 187: 153–160. 8238963 10.1007/BF00171747

[23] Kume T, Jiang H, Topczewska JM, Hogan BL (2001) The murine winged helix transcription factors, Foxc1 and Foxc2, are both required for cardiovascular development and somitogenesis. Genes Dev 15: 2470–2482. 11562355 10.1101/gad.907301 PMC312788

[24] Laclef C, Hamard G, Demignon J, Souil E, Houbron C, et al (2003) Altered myogenesis in Six1-deficient mice. Development 130: 2239–2252. 12668636 10.1242/dev.00440

[25] Relaix F, Rocancourt D, Mansouri A, Buckingham M (2005) A Pax3/Pax7-dependent population of skeletal muscle progenitor cells. Nature 435: 948–953. 15843801 10.1038/nature03594

[26] Petropoulos H, Gianakopoulos PJ, Ridgeway AG, Skerjanc IS (2004) Disruption of Meox or Gli activity ablates skeletal myogenesis in P19 cells. J Biol Chem 279: 23874–23881. 15039437 10.1074/jbc.M312612200

[27] Ridgeway AG, Skerjanc IS (2001) Pax3 is essential for skeletal myogenesis and the expression of Six1 and Eya2. J Biol Chem 276: 19033–19039. 11262400 10.1074/jbc.M011491200

[28] Heanue TA, Reshef R, Davis RJ, Mardon G, Oliver G, et al (1999) Synergistic regulation of vertebrate muscle development by Dach2, Eya2, and Six1, homologs of genes required for Drosophila eye formation. Genes Dev 13: 3231–3243. 10617572 10.1101/gad.13.24.3231 PMC317207

[29] Wilm B, James RG, Schultheiss TM, Hogan BL (2004) The forkhead genes, Foxc1 and Foxc2, regulate paraxial versus intermediate mesoderm cell fate. Dev Biol 271: 176–189. 15196959 10.1016/j.ydbio.2004.03.034

[30] Zhang C, Basta T, Fawcett SR, Klymkowsky MW (2005) SOX7 is an immediate-early target of VegT and regulates Nodal-related gene expression in Xenopus. Developmental Biology 278: 526–541. 15680368 10.1016/j.ydbio.2004.11.008

[31] Savage J, Conley AJ, Blais A, Skerjanc IS (2009) SOX15 and SOX7 differentially regulate the myogenic program in P19 cells. Stem Cells 27: 1231–1243. 19489079 10.1002/stem.57

[32] Bryson-Richardson RJ, Currie PD (2008) The genetics of vertebrate myogenesis. Nat Rev Genet 9: 632–646. 18636072 10.1038/nrg2369

[33] Weintraub H, Davis R, Tapscott S, Thayer M, Krause M, et al (1991) The myoD gene family: nodal point during specification of the muscle cell lineage. Science 251: 761–766. 1846704 10.1126/science.1846704

[34] Buskin JN, Hauschka SD (1989) Identification of a myocyte nuclear factor that binds to the muscle- specific enhancer of the mouse muscle creatine kinase gene. Mol Cell Biol 9: 2627–2640. 2761542 10.1128/mcb.9.6.2627-2640.1989 PMC362335

[35] Murre C, McCaw PS, Baltimore D (1989) A new DNA binding and dimerization motif in immunoglobulin enhancer binding, daughterless, MyoD, and myc proteins. Cell 56: 777–783. 2493990 10.1016/0092-8674(89)90682-x

[36] Blomhoff R, Blomhoff HK (2006) Overview of retinoid metabolism and function. J Neurobiol 66: 606–630. 16688755 10.1002/neu.20242

[37] Edwards MK, Harris JF, McBurney MW (1983) Induced muscle differentiation in an embryonal carcinoma cell line. Mol Cell Biol 3: 2280–2286. 6656767 10.1128/mcb.3.12.2280-2286.1983 PMC370099

[38] Tolkunova EN, Fujioka M, Kobayashi M, Deka D, Jaynes JB (1998) Two distinct types of repression domain in engrailed: one interacts with the groucho corepressor and is preferentially active on integrated target genes. Mol Cell Biol 18: 2804–2814. 9566899 10.1128/mcb.18.5.2804 PMC110659

[39] Yu JY, DeRuiter SL, Turner DL (2002) RNA interference by expression of short-interfering RNAs and hairpin RNAs in mammalian cells. Proc Natl Acad Sci U S A 99: 6047–6052. 11972060 10.1073/pnas.092143499 PMC122899

[40] Gianakopoulos PJ, Mehta V, Voronova A, Cao Y, Yao Z, et al (2011) MyoD directly up-regulates premyogenic mesoderm factors during induction of skeletal myogenesis in stem cells. J Biol Chem 286: 2517–2525. 21078671 10.1074/jbc.M110.163709 PMC3024746

[41] Voronova A, Al Madhoun A, Fischer A, Shelton M, Karamboulas C, et al (2012) Gli2 and MEF2C activate each other’s expression and function synergistically during cardiomyogenesis in vitro. Nucleic Acids Res 40: 3329–3347. 22199256 10.1093/nar/gkr1232 PMC3333882

[42] Livak KJ, Schmittgen TD (2001) Analysis of relative gene expression data using real-time quantitative PCR and the 2(-Delta Delta C(T)) Method. Methods 25: 402–408. 11846609 10.1006/meth.2001.1262

[43] Brannon M, Kimelman D (1996) Activation Of Siamois By the Wnt Pathway. Developmental Biology 180: 344–347. 8948596 10.1006/dbio.1996.0306

[44] Ovcharenko I, Loots GG, Giardine BM, Hou M, Ma J, et al (2005) Mulan: multiple-sequence local alignment and visualization for studying function and evolution. Genome Res 15: 184–194. 15590941 10.1101/gr.3007205 PMC540288

[45] Kemler R (1993) From cadherins to catenins: cytoplasmic protein interactions and regulation of cell adhesion. Trends Genet 9: 317–321. 8236461 10.1016/0168-9525(93)90250-l

[46] Grifone R, Demignon J, Giordani J, Niro C, Souil E, et al (2007) Eya1 and Eya2 proteins are required for hypaxial somitic myogenesis in the mouse embryo. Developmental Biology 302: 602–616. 17098221 10.1016/j.ydbio.2006.08.059

[47] Matt N, Dupe V, Garnier JM, Dennefeld C, Chambon P, et al (2005) Retinoic acid-dependent eye morphogenesis is orchestrated by neural crest cells. Development 132: 4789–4800. 16207763 10.1242/dev.02031

[48] Lagha M, Brunelli S, Messina G, Cumano A, Kume T, et al (2009) Pax3:Foxc2 reciprocal repression in the somite modulates muscular versus vascular cell fate choice in multipotent progenitors. Dev Cell 17: 892–899. 20059958 10.1016/j.devcel.2009.10.021

[49] Voronova A, Coyne E, Al Madhoun A, Fair JV, Bosiljcic N, et al. (2012) Hedgehog signalling regulates MyoD expression and activity. J Biol Chem., doi:10.1074/jbc.M112.400184. 10.1074/jbc.M112.400184 PMC3567689 23266826

[50] Kim CH, Neiswender H, Baik EJ, Xiong WC, Mei L (2008) Β-catenin interacts with MyoD and regulates its transcription activity. Mol Cell Biol 28: 2941–2951. 18316399 10.1128/MCB.01682-07 PMC2293083

[51] Ridgeway AG, Petropoulos H, Wilton S, Skerjanc IS (2000) Wnt signaling regulates the function of MyoD and myogenin. J Biol Chem 275: 32398–32405. 10915791 10.1074/jbc.M004349200

[52] Shi DL, Bourdelas A, Umbhauer M, Boucaut JC (2002) Zygotic Wnt/β-catenin signaling preferentially regulates the expression of Myf5 gene in the mesoderm of Xenopus. Dev Biol 245: 124–135. 11969260 10.1006/dbio.2002.0633

