# Supplementary material for: β-catenin Is Essential for Efficient In Vitro Premyogenic Mesoderm Formation but Can Be Partially Compensated by Retinoic Acid Signalling
Source: PLoS One. 2013 Feb 27;8(2):e57501. doi: 10.1371/journal.pone.0057501 (PMC3583846; doi:10.1371/journal.pone.0057501)
Supplement: Figure S1 — RA enhanced the expression of MRFs and MHC in P19[shControl] cells. P19[shControl] cells were differentiated in the presence of 1% DMSO with or without 3 nM RA as described in Materials and Methods. RNA was harvested from day 0 (undifferentiated) and day 9 (differentiated) cells and analyzed using RT-QPCR to quantify the transcript levels of the indicated genes. Data was normalized to β-actin, calculated as fold change relative to day 0 cells and expressed as percent maximum. Error bars indicate +/− SEM. The Student’s t-test was used to assess statistical significance, where **p-value<0.01, n = 3. (PDF) [file pone.0057501.s001.pdf]

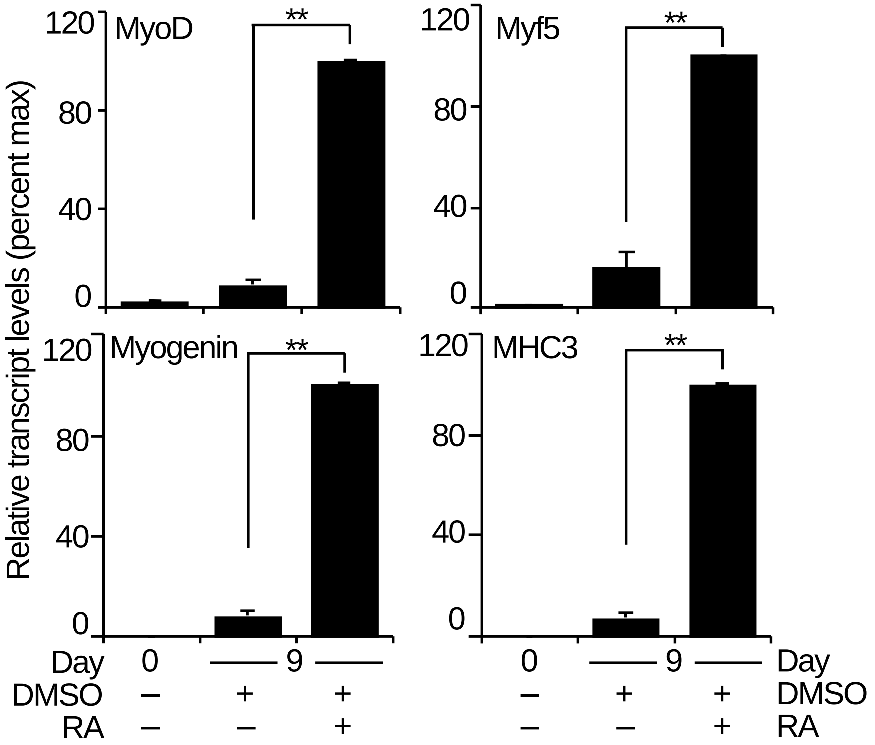

Figure S1. RA enhanced the expression of MRFs and MHC in P19[shControl] cells. P19[shControl] cells were differentiated in the presence of 1% DMSO or 1% DMSO and 3nM RA as described in Materials and Methods. RNA was harvested from day 0 (undifferentiated) and day 9 (differentiated) cells and analyzed using RT-QPCR to quantify the transcript levels of the indicated genes. Data was normalized to beta-actin, calculated as fold change relative to day 0 cells and expressed as percent maximum. Error bars indicate +/- SEM. The Student's t-test was used to assess statistical significance, where **p-value<0.01, n=3.
